# Developing Drugs for Children and the Adjustment of Medication—Is It a New Challenge or an Adaptation of Past Ideas?

**DOI:** 10.3390/jpm1010005

**Published:** 2011-12-06

**Authors:** Pascale Gauthier, Jean-Michel Cardot

**Affiliations:** University Clermont 1, UFR Pharmacie, Biopharmaceutical Department, 28, Place H. Dunant, B.P. 38, F-63001 Clermont-Ferrand, France; E-Mail: J-Michel.CARDOT@u-clermont1.fr

**Keywords:** pediatric drugs, oral administration, design

## Abstract

Nowadays the adjustment of medication for each patient is at the center of health strategy. Children can be considered as specific targets with their own specificities. In the oral route field some examples of drugs especially adapted to children can be found. Design is introduced in drug formulation to offer a better choice of products and now, children can be considered as partners in their own treatment. Enhanced comprehension of children's requirements can also lead to creation of drugs that improve compliance.

## 1. Introduction

Medicine and Pharmacy have seen a huge evolution in the past century. One hundred years ago, medicinal products were formulated and prescribed using a centesimal formula by the medical doctor and prepared specially by the pharmacist for a specific patient. This allowed adjustment of the dose, route, and type of administration, formulation and even taste for each patient. With the natural evolution of techniques, the pharmacy has become more industrialized. This has allowed a homogenization of the formulation and presentation, a greater certitude of the doses administered, an increase and a constancy of the quality, an increase of the productivity, reproducibility and a decrease in the risks linked with inaccurate preparation. This has also resulted in a decrease in price, leading to a greater access to medication for all the population. However this modification in the structure of production of drugs has led to a modification of prescription habits and removed the possibility of fine tuning the prescription for each patient, even though overall a great benefit for the global health has been achieved.

Nowadays the adjustment of the medications (and of the prescription) for each patient is again at the center of health strategies for some specific population groups such as children, the elderly and for some severe illnesses such as cancer and transplants. This paper focuses on how dispensing devices and design of the medicinal products could help special population groups such as pediatrics, increase compliance and achieve better treatments. However the industry cannot prepare drugs for each patient, and in this paper, some devices and ideas developed to help children's treatments and their compliance are presented.

## 2. Discussion—Presentation of Problems

In Europe the population below 18 years corresponds to 24% of the whole European population [1]. For a long time the 120 million European children have been neglected by health systems and we have described children in only two groups: new born (from 0 to 1 month) and children from 1 month to 15 years, so clearly there has been a lack of age differentiation! But in fact, depending on countries and use, newborns, infants, toddlers and children are all good terms that can be used and found and often associated with pharmacological studies; however similar terminologies cannot always be associated with similar ages [2]. Different authors [3–5] observe the following progression: preterm neonates (<36 weeks gestation, 0–27 days) full-term neonates or newborn (0–27 days) infants and toddlers (28 days–23 months), children (2–11 years) adolescents (12–17 years).

More than 80% of child populations are represented by children over 2 years old. The larger category, representing around 50% of all children, is composed of preschool and school children. This classification is very important as age is a key factor in the developmental status of different organs and physiological functions. As children form a heterogeneous group, drug development must be done as a function of age. In the past, children were often regarded as ‘little adults’ and therefore received a simple adaptation of adult dosages. Only recently, special attention and laws have been set up in USA and in Europe in the form of pediatric acts associated with financial incentives, and the European Medical Agency (EMA) has published a list of 400 priority drugs with unmet needs in children [3,6,7].

The main input of design in the pharmaceutical field is often centered on creation of a product that could be functional in all areas of everyday life with improved compliance. However a drug is a specific substance and medicinal product, a specific consumer product. In everyday life, we must not forget that the real user of the drug, the patient, the ill person, is always placed at the end of the health chain [8–10]. This user (the child in this case) is often different from the person who gives the drug (parents) and different from the doctor (who fills the first role in medical consultation, diagnosis and prescription) and different from the pharmacist, who has a second advisory role in the issue of the prescription. Parents play a very important role in this and we can describe a triangular relationship between children, health workers and parents. Parents have to help children to understand and accept their treatment and they have also to manage their own stress associated with the child's illness [5,8–10].

Age is a key factor, for example enzymatic systems, like cytochromes, become mature after two years modifying, of course, all pharmacokinetic aspects. Dose is adjusted following closely changes in weight and is frequently quoted as amount/Kg. This has led to formulations which could be adapted to this dose prescription [11,12]. Another important factor is concerned with food and alimentary habits. With age, the child passes from a liquid to solid diet and the digestive system becomes mature. The administration of drugs for newborns or infants might be possible, with oral formulations, with liquids such as milk or fruit juice. Another aspect to take into account is the swallowing capacity of the children which is related to the type of formulation that can be used [4,5,13,14]. The other important point, concerned with the age of children, is difficulty in their expressing feelings about their illness and the acceptance of the drugs as well the associated side effects. As a consequence, pain was mostly underestimated for a long time in newborns and young children.

The formulation and route of administration depended on the autonomy and age. Usually (except for some illnesses such as diabetes for example) the injectable route is restricted to hospitals due to the need for an MD or nurse. Rectal administration is simple and presents low risks but is limited by its acceptability which is linked to the age of the patient. Oral solid dosage forms may present a higher risk in young patients, and solid or liquid formulation may exhibit a poor taste compliance problem. The European Medical Agency has published recommendations [6].

## 3. Review and Example of Solutions Proposed in Oral Administration

### 3.1. Taste of Drugs

Preferences of children in term of taste are quite fundamentally different from those of adults. Classically sweet candy flavors are preferred by children [5,7,15]. As the original taste of active pharmaceutical ingredients and fillers can be unacceptable, the EMA has published a list of recommended flavors depending on the original taste of Active Pharmaceutical Ingredient (API) (acid, alkaline, bitter, salty, and sweet). There is also a proposal for flavors usually associated with treatment of different disease (vitamins and lemon/orange, stomach pain and mint flavors, *etc*.) [3,6].

Cultural factors also have to be taken into account, even if a company cannot produce the same medicinal products with different tastes according to country preferences. The simplest example from the sweet industry is the liquorice taste which is a ‘must’ in Nordic countries [1] where, for example, some salty liquorice ‘sweets’ exist under various brand names and are called “salmiak” or “salmiakki”.

### 3.2. Drug Administration Techniques for Children

Generally even if there is no ideal formulation, for children the following considerations must be taken into account: a minimal dosage frequency must be chosen, and normally one dosage form must fit all or a full range of products; developers must present a drug that has a minimal impact on life style with minimum toxic excipients. The drug must also be convenient, easy to use, with reliable administration. It must be easily produced, with a good stability and it must have low cost and a good commercial viability [1]. Oral administration can be achieved via several types of dosage forms; generally for children less than 6 years, oral solutions or suspension are commonly used. We can also find oral flexible dosage form presented as granules, pellets or powders that allow an easy reconstitution in solution/suspension and avoid any problems of swallowing for the younger children [1,3,4,6,7,16].

Different objectives can be defined. The first one is to help parents to administer medicines, another is to help parents make treatment acceptable to children, and lastly to help children take their own medicine and increase their autonomy. Depending on the age, these factors are different. For example for babies and newborns a passive administration is the norm [5,9,10]. They do not know what a drug is; drugs that can be administered are presented as powders, solutions or suspensions and some are prepared and mixed by parents with the baby's milk, sometimes with added sugar. When the children become older, the adults can try to explain and convince them to take a treatment/themselves.

Some alternatives have been developed such as active management: nipples, pacifiers or lollipops with double use (child can play them while absorbing a dose of medication) [5]. The suppliers, as well as parents, must be warned because systems must be totally different from the original and exhibit an obvious difference so as to avoid any confusion or over consumption [10]. For example, strips and ibuprofen chewing-gum actually exist in USA for pain killer administration.

### 3.3. Metering Systems for Oral Administration in Children

**Figure 2:**
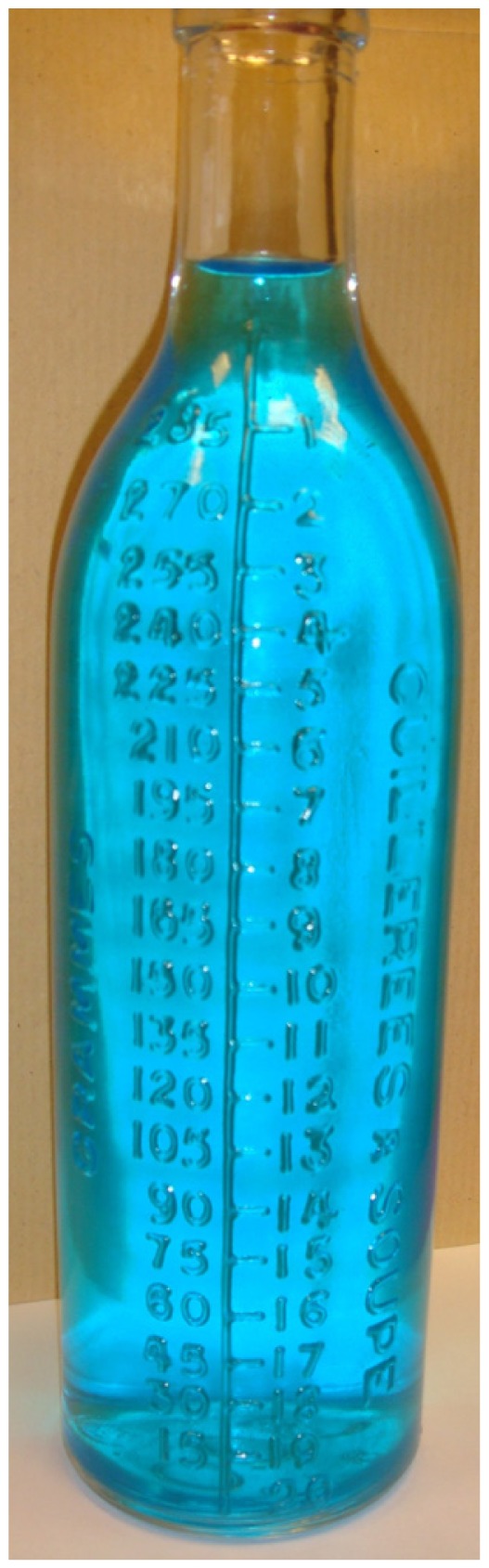
Example of glass bottle showing spoons measurements.

Following age and weight specificities, the product must be dosed correctly. From the beginning of the century up to the 1950's, specific glass bottles showing spoons measurements were used allowing an accurate dosage of product (Figure 2). This system was available and useful for preparations made directly by the pharmacist.

**Figure 3:**
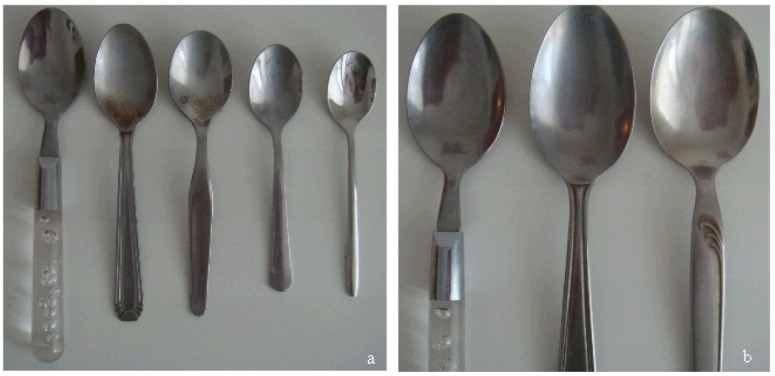
Example of different spoons household (a: tea spoons; b: table spoons).

Following industrialization, this was abandoned and replaced by household spoons up to the 1980's but for pediatric solutions or suspensions, the use of spoons or half spoons led to specious accuracy as it appears that there are considerable differences (Figure 3) within households. Even if experts can say or claim that there is a low risk of harm due to these differences, the accuracy of dosage with these spoons is not good.

**Figure 4:**
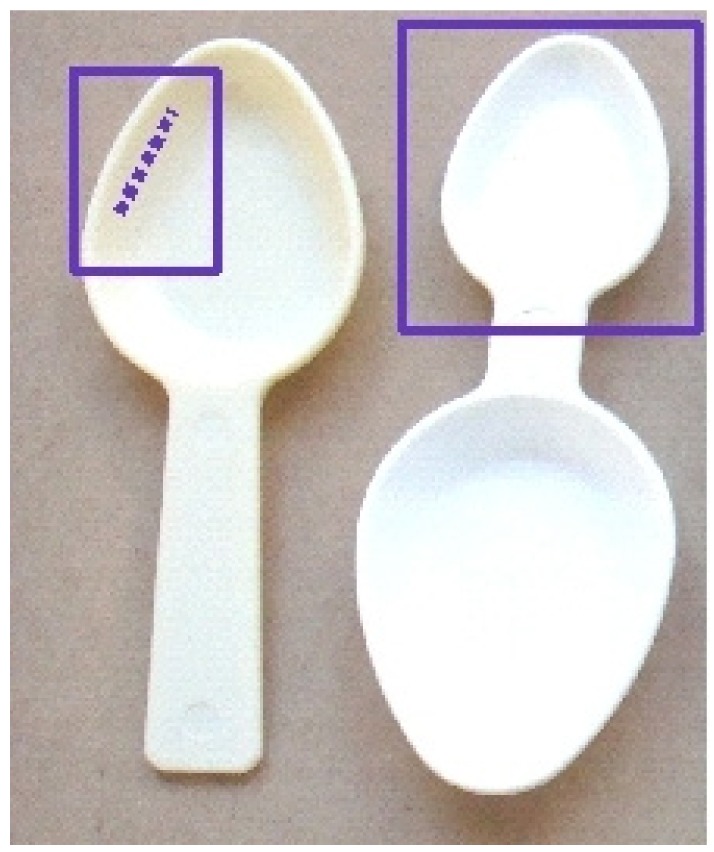
Example of full or half volume spoons (in purple square).

The idea of standardization was revisited with adaptations of specific spoons or mini glasses included in the medication boxes with different systems. The first type of system still comprised of spoons with fixed volumes corresponding to a full or half a spoon (Figure 4).

The second generation of devices was developed based on concepts of having a simpler accurate and reliable dosing. It could be considered as a rupture in the prescription strategy.

**Figure 5:**
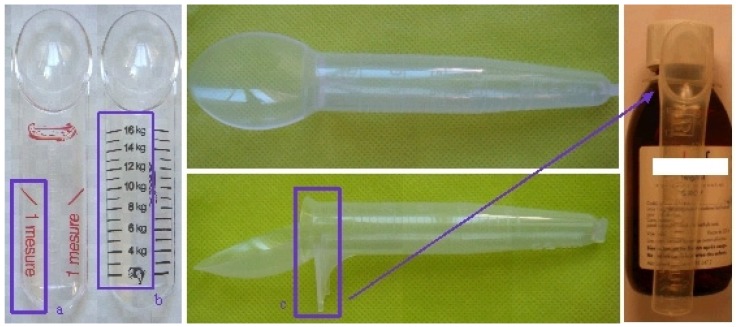
Examples of different M-T-spoons^®^ (a: dose by spoon; b: dose by kg; c: spoon attached to the bottle).

It is based on a simplification of their use by the parents involving a modification of the protocol prescribed by the medical doctors based on kg of body weight. For example the M-T-Spoon^®^ that offers different forms of ‘spoon like’ measurements (Figure 5a) or a reliable dose corresponding to the weight of the children in kg (Figure 5b). Some spoons are clipped directly attached to the bottle and allow the correct dosing system and the correct drug (Figure 5c).

**Figure 6:**
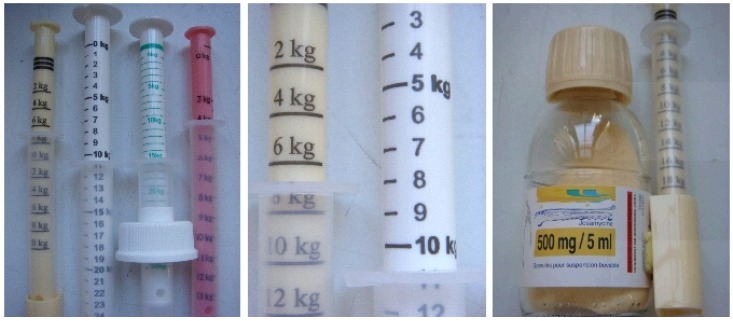
Examples of different syringes used for dosing pediatric suspensions.

The next step was the modification from a passive tool to a more active one, involving not only the action of filling a reservoir but pulling the plunger of a syringe, the bottle being in this case safely on the table avoiding any risk of being knocked over. The first example of this system was the Adapta-dose^®^ syringe that measures a prescription of emulsion, solution or suspension according to the weight of children (Figure 6). All these systems can help to deliver the right dose but clear instructions should be provided (e.g., pictograms on secondary packaging) on the correct filling of the syringe (to avoid any air bubbles, *etc*.). The graduation of the oral syringe must be appropriated to avoid any confusion (use of mg can be a problem) [4]. Some countries, such as France, propose the use of kg as units of measuring relating to the weight of patient and these new metering systems led pediatricians to change their prescriptions for children; it is now done by body mass of children and not by mg of drug. This allows better matching of treatment to the real user, the child according to his weight.

All these packaging and metering systems are easy to use and present good accuracy. However, some problems can appear with dosing syringes as reported by some health authorities because pressure of delivery can sometimes lead to problems in swallowing [17]. This suggests the need for new systems with mandatory low pressure administration.

**Figure 7:**
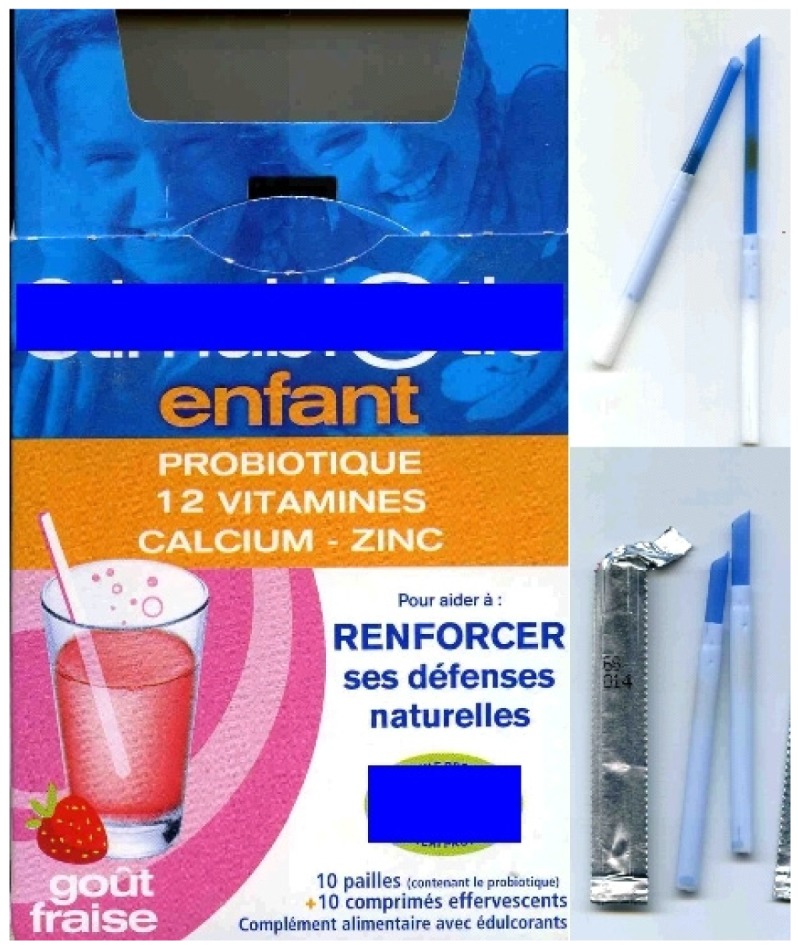
Example of straw used for probiotic suspension.

Other systems are also appearing for oral dosage and administration. For example, in 2001, a well known French designer, Matthieu Lehanneur, has proposed ‘therapeutic objects’ that use different systems to treat disease (they have now found their place in one of the most famous museums in the world, the Museum Of Modern Art—MOMA—in New York). One of these objects is ‘the sleep wand’ integrated with a straw to relieve problems of insomnia. In this case the technique involves infusing a 'wand in a glass of water and waiting for it to soften before drinking the mixture [18]. Industrially the idea of the straw has been employed, as a conservation and dispensing device. One example of such a product is associated with probiotics contained in the straw which are added to an effervescent tablet of vitamins (Figure 7) just before administration.

New straw devices are appearing as safe dosing systems for different API in granulates. The straw allows storage of products with also a reliable measure of the dose and generally children love to drink liquid with a straw. The use of the straw helps in drinking thicker liquids and is fun for children [19]. With this new system, the medicine can then be either administered by smoothly shooting the straw into the patient's mouth (passive administration) or by adding it to a drink and sipping it through the syringe (active administration) [4,19].

**Figure 8:**
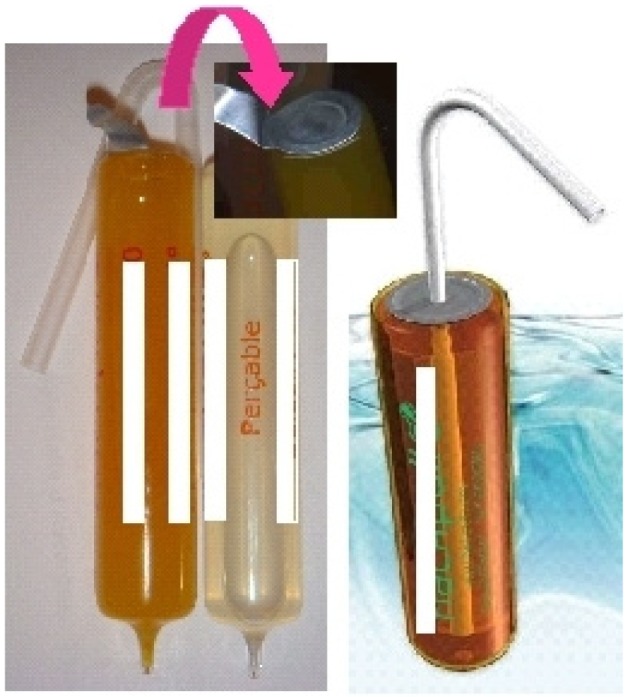
Glass vial and straw [20].

Another example is the old glass vial, redesigned and associated with a straw to give a new system that can be easily employed with all API requiring a glass for stability. In this case, the vial is employed in a glass single dose system and it is opened with a peel back lid and drunk with a straw; different vial sizes are available [20] (Figure 8).

### 3.4. A Recurrent Problem: Avoiding Over and Mis-Consumption

Improving treatment and compliance presents a difficult challenge especially for children that need to take their drugs only when they are ill. In fact, improving treatment can also be a paradox especially for children, because helping them to take a drug must not lead to drug overconsumption if the drug starts to play a central role in a funny story or has a special good taste like a sweet [10]. Offering children drugs with sweet taste, and packaging using a friendly design can help to improve compliance. Nonetheless drugs must have a special place in treating disease. Even though a pleasant taste can help children to take it easily, it must not encourage overconsumption. Further, an attractive design that can help children to like taking treatment must not lead children becoming addicted. On the other hand, for adults' treatments, childproof systems are being developed. Their objective is to limit the access by children to certain products thus avoiding accidents and overdosing. The best childproof systems may not fulfill their goal because children are always curious, and integrating childproof systems must not mean creation of products impossible to use by parents or grandparents.

**Figure 9:**
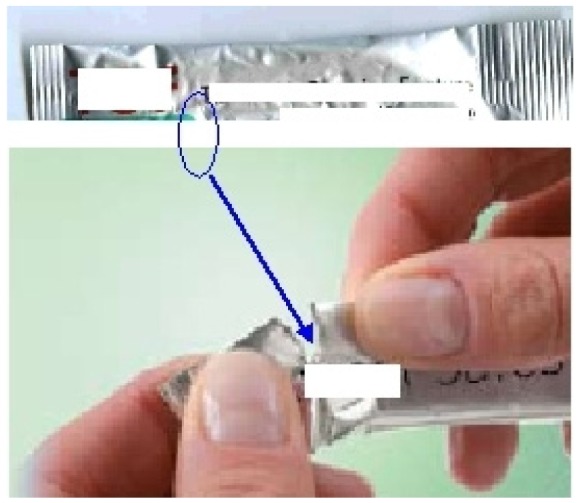
Child-resistant SafeStick™ tear-open pack [21].

New materials are also leading to new ideas such as for example a novel child resistant stick with an easy tear open pack incorporating a printed tear initiation point. The SafeStick™ tears [21] easily but only in a special defined area, presenting a controlled unit dosing system with a child resistant format incorporating unidose systems for liquid forms that can be safely used (Figure 9).

### 3.5. Drugs for Children and Designer Work

**Figure 10:**
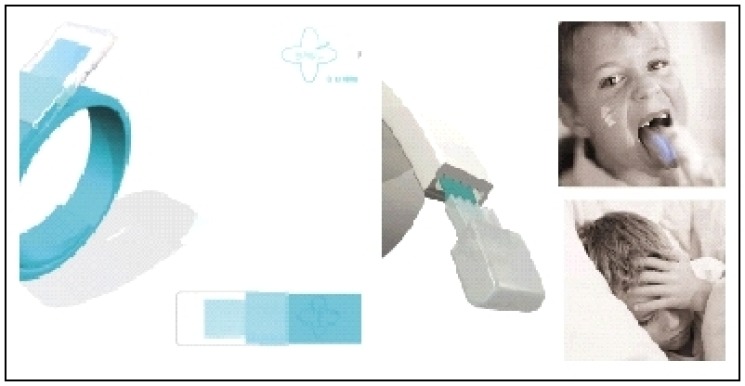
Strip with paracetamol (length of the strip corresponds to the dose).

As an example, Anne-Charlotte Legrand, a young designer has chosen to present a new approach for targeting children between 3 and 6 years old. She focused on three minor pathologies very frequent in childhood: injuries, fever and pain, and pulmonary problems. The designer has presented a range of different products named Tamalou^®^ that need a preparation time considered as a medical digression during which the child will participate in the medication. This moment can be between the eating times and play time and it can be considered as a learning time (for better adherence) which allows for dialogue to explain the disease. Further, we can limit the passivity of children when confronted with their illness and explain to them the importance of taking care of themselves. Each product operates following the same pattern: mother prepares and explains things to the child (often with a nice story presented in a booklet), and once the system is prepared, she entrusts the device to the child so that he or she can participate actively in the care [22,23]. For example a paracetamol strip, a system which allows dispensing the strip in centimeters cut according to body weight. The choice of length is directly related to the concept of graduation syringe dosage used with liquid form. In that case designer's work is adapted to the solid dosage form (Figure 10).

Two other Tamalou's devices are proposed with a similar idea for preparation by the parent before giving it to the child. One is a felt, is impregnated with antiseptic swabs where the parent prepares the buffer and applies it as a magic wand. The last one is a booklet with pictures impregnated with a balm with essential oils to sooth the bronchial passage that relieves symptoms (e.g., for a cold…) and tells a story [22,23].

These systems have been developed in order to allow the child to be involved as a player in their treatment because work is also in direct relation to the child partner hypothesis where the child can be involved as a player in the observance of his treatment because a drug well taken will be more efficient.

## 4. Conclusion

These different examples (not a full review) can show that in pediatric areas more developments have appeared during the ten last years. Now, children are considered as specific patients with their own specific characteristics and more and more developments have appeared in the pharmaceutical area. Building a ‘child partner’ relationship is the best way forward taking the child as a real partner to fight disease, involving the child with all the other partners, doctors, parents, medication. One key point is to invite children to participate and understand the outcome of the treatment with the help of their parents and doctors. Another key factor is the importance and accuracy of the right dose, using the best administration device to avoid any risk of overdosed children. These conclusions are close to those of a century ago when the prescriptions were made specifically for each patient. However, it must be kept in mind that even if the ‘appeal’ is successful, the pharmaceutical products must not be so attractive as to cause over or mis-consumption.

## Figures and Tables

**Figure 1:**
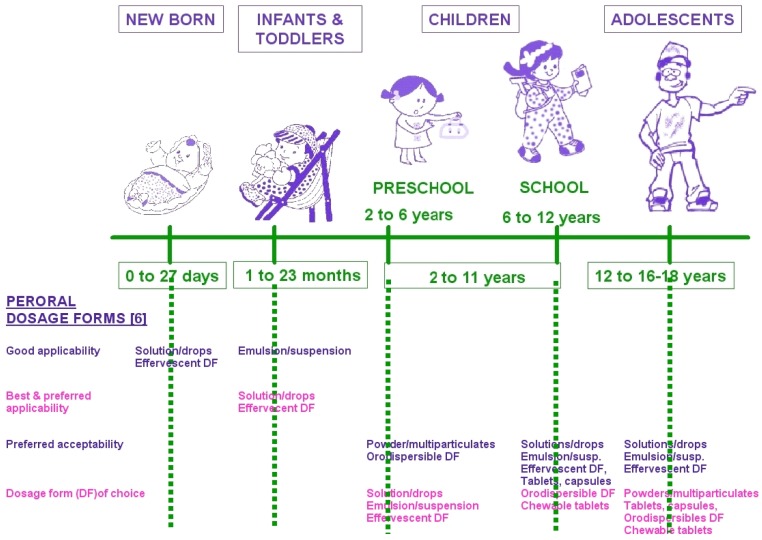
Age groups within the pediatric population and indication of peroral dosage forms applicability and preferences per age [3–6].

**Figure 11:**
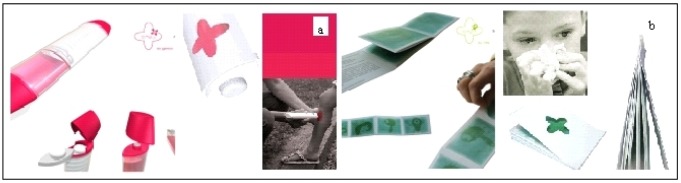
Antiseptic as a magic wand (a) and Balm with essential oil (b).
